# Empirical antifungal treatment in the critically ill patients: how does it impact on the outcome?

**DOI:** 10.1186/cc10654

**Published:** 2012-03-20

**Authors:** R Bruyère, C Vigneron, J Quenot, M Hamet, F Dalle, S Prin, PE Charles

**Affiliations:** 1 University Hospital, Dijon, France

## Introduction

Given the high mortality attributable to invasive candidiasis (IC) and the lack of reliable diagnosis tool, antifungals are often started in high-risk patients with severe sepsis despite the absence of proven disease. According to current guidelines, echinocandins are the drugs of choice in this setting. However, the level of evidence supporting this statement is low.

## Methods

A retrospective single-centre observational study including every patient with highly suspected but unproven IC (that is, Candida score >3, multifocal *Candida *sp. colonization, unresolved sepsis despite >2-day broad-spectrum antibiotics, negative blood culture) who received at least two doses of one echinocandin between 2008 and 2011. Patients with proven IC (that is, candidemia) over the same period were used as controls. These two groups of patients were compared regarding baseline characteristics and both clinical and biological follow-up data while receiving antifungal therapy. The clinical response to antifungal therapy was assessed through the SOFA score daily decrease from day 0 to day 3 in both groups and compared by repeated-measures ANOVA. Then, independent predictors of death in the ICU were determined by Cox regression analysis.

## Results

Fifty-one patients were included (30 with suspected IC and 21 with proven IC). At the onset of antifungal therapy, the Candida score was greater in the patients with suspected IC than in those with proven infection (3.7 ± 0.7 vs. 3.0 ± 0.8, *P *= 0.001) since multifocal colonization was more frequent in the former. In addition, the patients with suspected but unproven IC looked more seriously ill according to the SOFA score (8.3 ± 3.0 vs. 6.6 ± 3.5, *P *= 0.07). This mainly resulted from a greater level of hypotension as assessed through the SOFA score (2.8 ± 1.5 vs. 1.2 ± 1.5 points, *P *= 0.0006). Obviously, the clinical response to antifungal therapy was significantly more consistent in the patients with unproven IC than in those with proven infection (*P *= 0.032). In addition, there was a trend toward an improved survival in the former patients (53 vs. 47%, *P *= 0.42). The only independent protective factor was echinocandin therapy duration (HR = 0.84 (95% CI 0.75 to 0.94), *P *= 0.0034).

## Conclusion

A significant clinical improvement is achieved in patients with suspected but not proven IC receiving empirical antifungal therapy with an echinocandin. In contrast, the patients with proven IC are less responsive to therapy and are more likely to die in the ICU. Our data support the use of an echinocandin as empirical therapy in very high-risk patients.
